# Cortisol-treated zebrafish embryos develop into pro-inflammatory adults with aberrant immune gene regulation

**DOI:** 10.1242/bio.020065

**Published:** 2016-07-21

**Authors:** Ellen I. Hartig, Shusen Zhu, Benjamin L. King, James A. Coffman

**Affiliations:** MDI Biological Laboratory, Kathryn W. Davis Center for Regenerative Biology and Medicine, Salisbury Cove, ME, 04672, USA

**Keywords:** Chronic stress, Glucocorticoid, Developmental programming, Immune system, RNA-seq, Zebrafish

## Abstract

Chronic early-life stress increases adult susceptibility to numerous health problems linked to chronic inflammation. One way that this may occur is via glucocorticoid-induced developmental programming. To gain insight into such programming we treated zebrafish embryos with cortisol and examined the effects on both larvae and adults. Treated larvae had elevated whole-body cortisol and glucocorticoid signaling, and upregulated genes associated with defense response and immune system processes. In adulthood the treated fish maintained elevated basal cortisol levels in the absence of exogenous cortisol, and constitutively mis-expressed genes involved in defense response and its regulation. Adults derived from cortisol-treated embryos displayed defective tailfin regeneration, heightened basal expression of pro-inflammatory genes, and failure to appropriately regulate those genes following injury or immunological challenge. These results support the hypothesis that chronically elevated glucocorticoid signaling early in life directs development of a pro-inflammatory adult phenotype, at the expense of immunoregulation and somatic regenerative capacity.

## Abstract (precis)

**Summary:** Chronically elevated glucocorticoid stress signaling during early zebrafish development has long-term consequences that affect immune gene expression and regulation in adulthood.

## INTRODUCTION

Chronic psychosocial stress contributes significantly to a wide variety of public health problems endemic to the modern world, many of which are linked to chronic inflammation (Berk et al., 2013; Christian et al., 2006; Cohen et al., 2007; Pawelec et al., 2014; Radek, 2010). A growing body of epidemiological evidence indicates that individuals that experience chronic stress early in life – even prenatally – tend to develop a pro-inflammatory phenotype, and hence increased susceptibility to inflammatory disease and degenerative aging in adulthood (Cohen et al., 2012; Harris and Seckl, 2011; Howerton and Bale, 2012; Miller et al., 2009).­

The systemic stress response in humans is mediated by the glucocorticoid hormone cortisol, a steroid secreted by the adrenal cortex in response to adrenocorticotropic hormone (ACTH). The latter is released by the anterior pituitary in response to corticotropin-releasing hormone (CRH) emitted by the hypothalamus in response to stress signals from the brain. This system, known as the hypothalamus-pituitary-adrenal (HPA) axis, induces metabolic and other cell physiological changes directed at stress mitigation. Following acute stress, the system turns itself off by way of a negative feedback loop wherein receipt of cortisol in the hypothalamus and pituitary extinguishes emission of CRH and ACTH. Chronic psychosocial stress compromises this mechanism by continuously activating the HPA axis, leading to chronically elevated cortisol levels, and ultimately (at least in some cases) to glucocorticoid resistance (Jung et al., 2015; Maldonado Bouchard and Hook, 2014; Stark et al., 2001).

Cortisol and other corticosteroids exert their biological effects by binding and thereby activating the glucocorticoid and mineralocorticoid receptors, DNA sequence-specific transcription factors in the steroid receptor family (Gomez-Sanchez and Gomez-Sanchez, 2014; Kadmiel and Cidlowski, 2013). Owing to its higher affinity for cortisol, the mineralocorticoid receptor is more or less constitutively active, whereas the glucocorticoid receptor (GR) is activated under stressful conditions, and is largely responsible for inducing the stress-responsive gene expression program (Sorrells and Sapolsky, 2007). The GR regulates gene expression both directly, by binding glucocorticoid responsive DNA sequence elements within the *cis*-regulatory domains of genes, and indirectly, by binding other transcription factors such as NF-κB. Direct targets include genes required for the metabolic changes entailed by the stress response, whereas indirect targets include immune system genes activated by NF-κB, which is inhibited by the GR, accounting in part for the well-known anti-inflammatory effects of glucocorticoids.

Elevated glucocorticoid signaling has epigenetic effects that are thought to mediate long-term developmental programming in response to early life adversity (Khulan and Drake, 2012). Consistent with this, chronic exposure to elevated corticosteroids during early development has been shown in a number of systems to have lasting phenotypic effects that can not only impact the health of the exposed individual, but also that of the individual's offspring (Drake et al., 2007). The epigenetic mechanisms for some of these effects have been elucidated, and include methylation of the GR promoter, resulting in lower GR expression and consequently glucocorticoid resistance (Chen et al., 2009; Tyrka et al., 2012; Weaver et al., 2004). However, the stress response system involving glucocorticoid signaling is complex and regulated at many levels, and the systemic effects of glucocorticoid-mediated developmental programming, including the global impact such programming has on somatic gene expression and regenerative capacity in the adult, is largely unknown.

The zebrafish is a premier model organism for studies of regeneration, owing to its remarkable ability to regenerate many of its body parts, including its tailfin, which provides a convenient assay for regenerative capacity. Studies of tailfin regeneration have begun to illuminate the genetic pathways required for regeneration, and it was recently shown that macrophages play an essential role in the process (Petrie et al., 2014), as has also recently been shown to be true for salamander limb regeneration (Godwin et al., 2013). Of interest in light of the results reported here, the GR is known to play a central role in macrophage regulation (Chinenov et al., 2014).

The neuro-endocrine stress response system is largely conserved between humans and zebrafish. As in humans, the primary stress hormone in zebrafish is cortisol, which is produced in the interrenal gland, the functional equivalent of the mammalian adrenal cortex. Zebrafish have recently emerged as a model for examining the role of glucocorticoid signaling in developmental programming (Nesan and Vijayan, 2013). In this study we used zebrafish to ask how exposure to chronically elevated cortisol during early development affects gene expression and regenerative capacity in adulthood.

## RESULTS AND DISCUSSION

### Glucocorticoid signaling is activated in zebrafish embryos exposed to exogenous cortisol

**Fig. 1.**
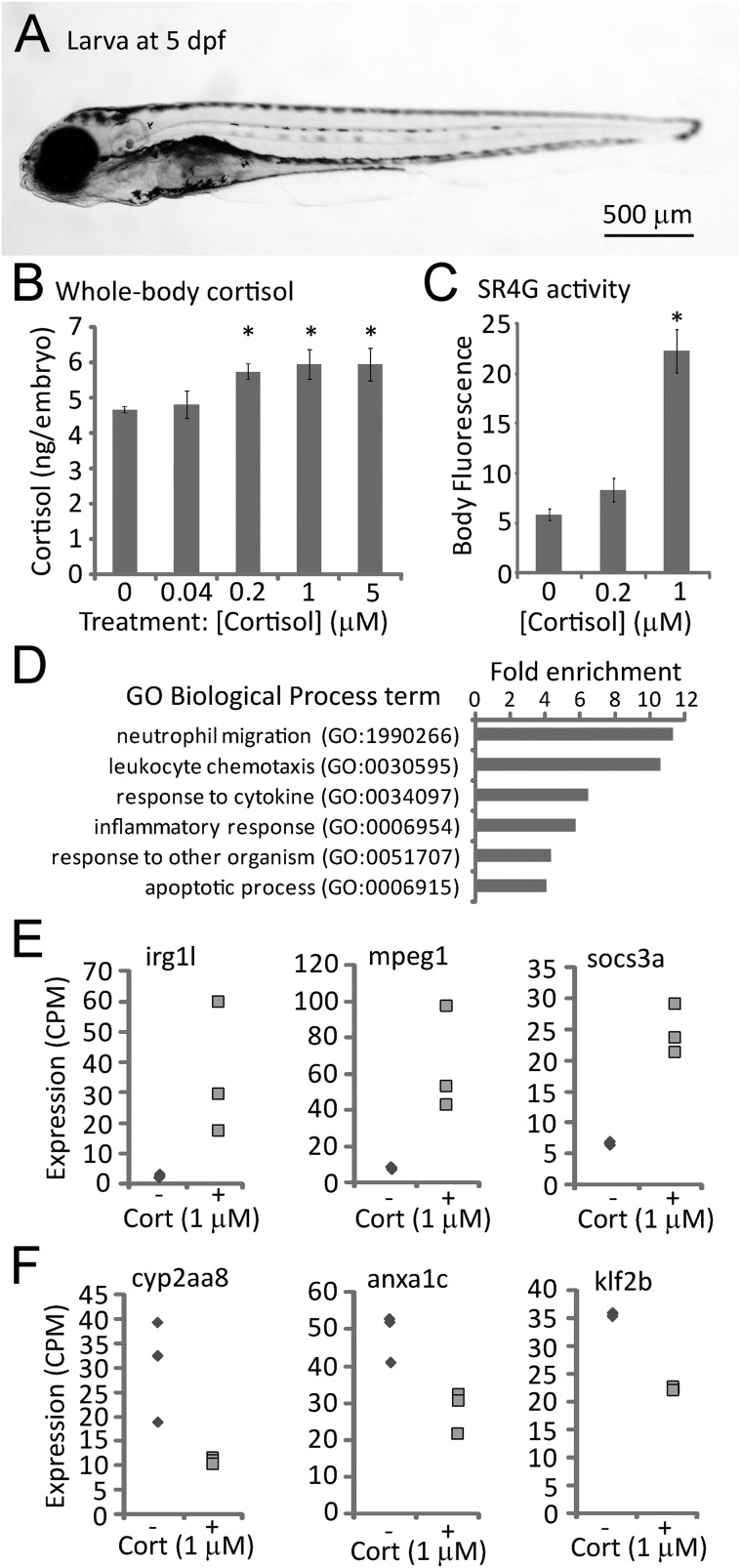
**Effects of cortisol treatment on zebrafish larval cortisol levels, glucocorticoid receptor activity and gene expression.** (A) Typical larva at 5 days post-fertilization (dpf). (B) Whole-body cortisol levels after exposure to varying doses of cortisol from 0-5 dpf, measured by ELISA of larval extracts (30 larvae per sample, duplicate measurements of each sample; *P*=0.015 by one-way ANOVA; * indicates that the difference from untreated controls is statistically significant by post hoc Tukey's test). (C) GFP fluorescence in the glucocorticoid receptor-responsive reporter line SR4G at 5 dpf following treatment with the indicated concentrations of cortisol (20 larvae imaged per sample; *P*=7.29×10^−11^ by a one way ANOVA; * indicates that the difference from untreated controls is statistically significant by post hoc Tukey's test). (D) Gene ontology biological process parent terms found by PANTHER to be significantly (*P*<0.05) overrepresented in the list of genes differentially expressed in 5 day old embryos treated with 1 μM cortisol. (E) RNA-seq measurements of three genes found to be upregulated in embryos treated (+) with 1 μM cortisol, compared to untreated controls (−). (F) RNA-seq measurements of three genes found to be downregulated in 1 μM cortisol-treated embryos (+) compared to untreated controls (−). Differences in expression between control and treatment groups shown in E and F are all statistically significant (*P*<0.05). Error bars in B and C depict standard error of the mean (s.e.m.).

Zebrafish embryos treated continuously with sub- to low-micromolar doses of exogenous cortisol from 0 to 5 days post-fertilization (dpf) were found to produce morphologically normal larvae (Fig. 1A), but with elevated whole body cortisol levels, as indicated by ELISA of larval extracts (Fig. 1B). The treated larvae upregulated known glucocorticoid-responsive genes (Fig. S1A), and larvae of a glucocorticoid-responsive GFP reporter line (SR4G, Krug et al., 2014) displayed elevated fluorescence in response to the treatment (Fig. 1C; Fig. S1B), indicating that the treatment activated the glucocorticoid receptor. Treated larvae had an elevated heart rate and increased levels of reactive oxygen species (Fig. S2), expected effects of stress signaling. We conclude from these results that zebrafish embryos treated with exogenous cortisol activate glucocorticoid receptor signaling and mount a systemic stress-response.

### Cortisol-treated larvae upregulate genes involved in immune system processes and the immune response

High-throughput sequencing of RNA (RNA-seq) extracted from larvae at 5 dpf was used to examine the genome-wide transcriptional effects of the cortisol treatment. Of the ∼20,000 expressed genes, 411 were found to be significantly (*P*<0.05) over-expressed and 144 were found to be significantly under-expressed in the cortisol-treated larvae. PANTHER overrepresentation analysis (Mi et al., 2013) of gene ontology (GO) biological process (BP) terms associated with the 555 genes that were significantly differentially expressed indicated a strong effect of the treatment on processes related to immune cell function and inflammation (Fig. 1D; Table S1). Similarly, GO BP term enrichment analysis using the GOrilla algorithm (Eden et al., 2009) to interrogate the full list of 9523 upregulated genes (i.e. all genes with relative expression treated/untreated >1) ranked by significance identified *immune system process* as the most significantly enriched BP term (Table S2), and visualization of all the significantly enriched BP terms (Supek et al., 2011) identified *immune response* and *cellular lipid catabolism* as the two largest parent categories of terms (Fig. S3A). Examples of significantly upregulated genes associated with immune response and/or regulation included *irg1l* (immunoresponsive gene 1-like; Hall et al., 2014), the macrophage marker *mpeg1* (Ellett et al., 2011), and the immunoregulatory gene *socs3a* (Qin et al., 2012) (Fig. 1E). GOrilla analysis of the 10,547 downregulated genes ranked by significance (Table S2) identified *membrane depolarization*, *cell communication*, and *long-chain fatty acid metabolism* (including *arachidonic acid metabolism*, known to regulate inflammation; Buckley et al., 2014) as the three largest parent categories of enriched BP terms (Fig. S3B). Examples of significantly downregulated genes associated with regulation and/or resolution of inflammation included *cyp2aa8* (Su and Ding, 2004), *anxa1c* (Vago et al., 2012), and *klf2b* (Nayak et al., 2013) (Fig. 1F). These data are consistent with known metabolic effects of elevated GR signaling (Sorrells and Sapolsky, 2007), but also suggest that prolonged activation of glucocorticoid receptor signaling during early development upregulates immune system gene activity. Since many immune system genes are responsive to the transcription factor NF-κB, we reasoned that NF-κB activity may be constitutively higher in cortisol-treated larvae. This was indeed the case, as shown by increased fluorescence of 5 day old cortisol-treated larvae of a transgenic NF-κB-responsive GFP reporter line (Kanther et al., 2011) (Fig. S4).

### Adults developed from cortisol-treated embryos have elevated basal cortisol and aberrant constitutive expression of genes involved in immune system response and regulation

**Fig. 2.**
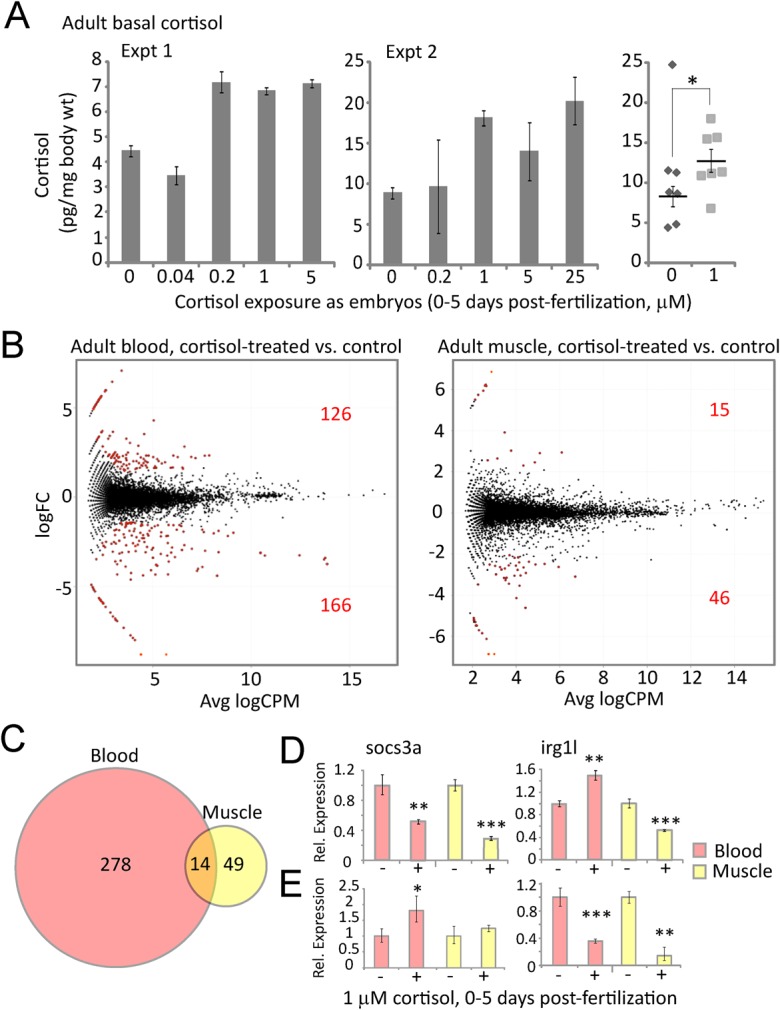
**Effects of early developmental exposure to cortisol on adult basal cortisol levels and gene expression in blood and muscle cells.** (A) Cortisol levels measured by ELISA of whole body extracts from individual 7-month-old adults derived from three experimental cohorts exposed as embryos to the indicated concentrations of cortisol from 0-5 dpf. Error bars in the first two graphs depict standard deviations (s.d.) for technical replicates; for the third graph showing combined data from all three experiments, the error bars represent s.e.m. for biological replicates. For the third experiment a power analysis was performed which determined that a minimum of five individuals was needed for statistically significant results based on the effect sizes obtained in the first two. Statistical significance *P=0.04 by a two-tailed *t*-test, excluding the single outlier (Q-test, Q_exp_=0.649>Q_crit_=0.569, *P*<0.05) in the control group. (B) Scatter plots of average expression level and fold-change in expression for each gene in blood and muscle tissue from 4.5-month-old adults derived from embryos treated with 1 μM cortisol and untreated controls. Red dots indicate genes with significantly different expression (*P*<0.05) between the two groups, the numbers of which (over- and under-expressed) are also shown in red. (C) Venn diagram showing numbers of differentially expressed genes in blood and muscle, and the overlap between the two (14 genes, listed in Table 1). (D,E) qRT-PCR measurements of *socs3a* and *irg1l* in blood and muscle tissue from cortisol treated embryos (+) and untreated control embryos (−) from (D) 9-month-old zebrafish from the same experimental cohort from which the RNA-seq results shown in B and C were obtained, and (E) 5-month-old zebrafish from a different experimental cohort. Statistical significance *P<0.05, **P<0.01, ***P<0.001 by an unpaired *t*-test, the error bars represent s.d.

The cortisol treatment regime did not significantly affect the rate that treated fish grew to adulthood (Fig. S5) and the adults derived from treated embryos displayed no obvious morphological or behavioral defects. However, adult fish derived from the treated embryos were found on average to have constitutively elevated basal cortisol levels (Fig. 2A), indicating that the treatment had a long-term activating effect on the stress system. To assess the long-term effects of the treatment on constitutive gene expression, RNA-seq was performed on RNA extracted from blood and muscle of 4.5-month-old adults derived from cortisol-treated larvae and their untreated (control) siblings. In both tissues more genes were found to be under-expressed than over-expressed in the fish derived from cortisol-treated embryos (Fig. 2B). GOrilla analysis (Tables S3 and S4) identified *leukocyte chemotaxis* as the most significant parent category for genes under-expressed in blood, and *response to external biotic stimulus* the largest parent category for genes under-expressed in muscle, with multiple subcategories in common between the two tissues, including *defense response*, *inflammatory response*, *response to chemical* and *response to external stimulus* (Fig. S6). Fourteen genes were significantly differentially expressed both in blood and muscle (Fig. 2C, Table 1), twelve of which were under-expressed in fish derived from cortisol-treated embryos. The latter included *irg1l* and *socs3a* (Table 1), two genes associated with immune system response and regulation that were among the most highly upregulated genes in the cortisol-treated larvae (see Fig. 1E). Quantitative reverse-transcription and polymerase chain reaction (qRT-PCR) confirmed that the latter two genes were differentially expressed in both blood and muscle of older adults derived from the same batch of treated embryos, although *irg1l* was found to be over-expressed rather than under-expressed in blood (Fig. 2D). The latter difference may reflect the fact that the fish used for the qRT-PCR analysis, while from the same experimental cohort as those used for RNA-seq, were significantly older (9 months). Indeed, in 5-month-old fish from another experimental cohort *irg1l* was again found to be significantly under-expressed in both blood and muscle (Fig. 2E). However, in these fish *socs3a* was not under-expressed, indicating that there is biological variability in the response of specific genes to the treatment. Despite this variability (not unexpected in wild-type fish), these results suggest that short-term treatment of zebrafish embryos with exogenous cortisol has long-term effects on the constitutive (basal) activities of both the stress and immune systems.

**Table 1.**
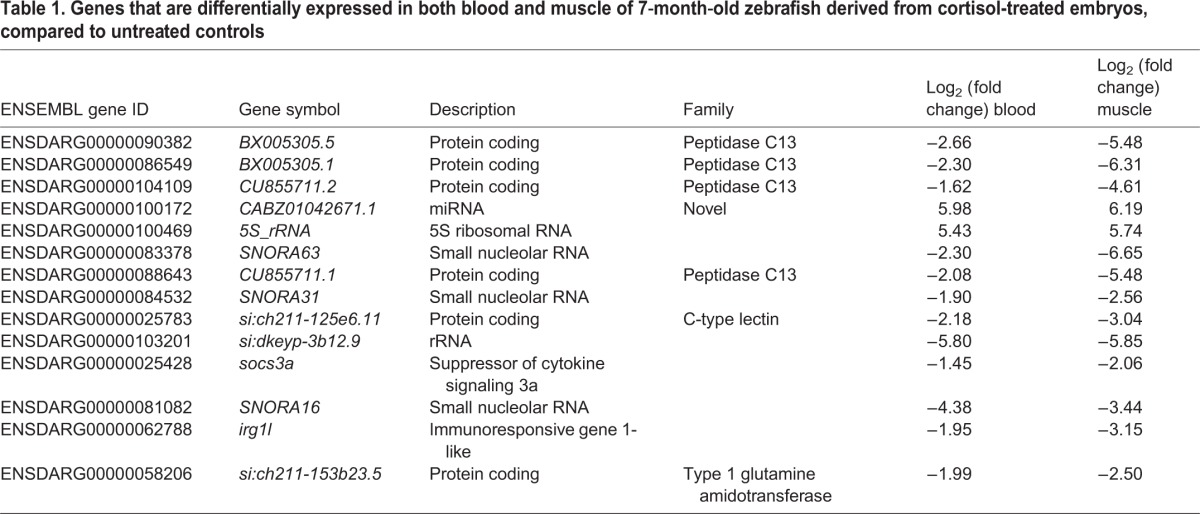
**Genes that are differentially expressed in both blood and muscle of 7**-**month**-**old zebrafish derived from cortisol-treated embryos, compared to untreated controls**

### Adults developed from cortisol-treated embryos have tailfin regeneration defects including aberrant expression of inflammatory genes

**Fig. 3.**
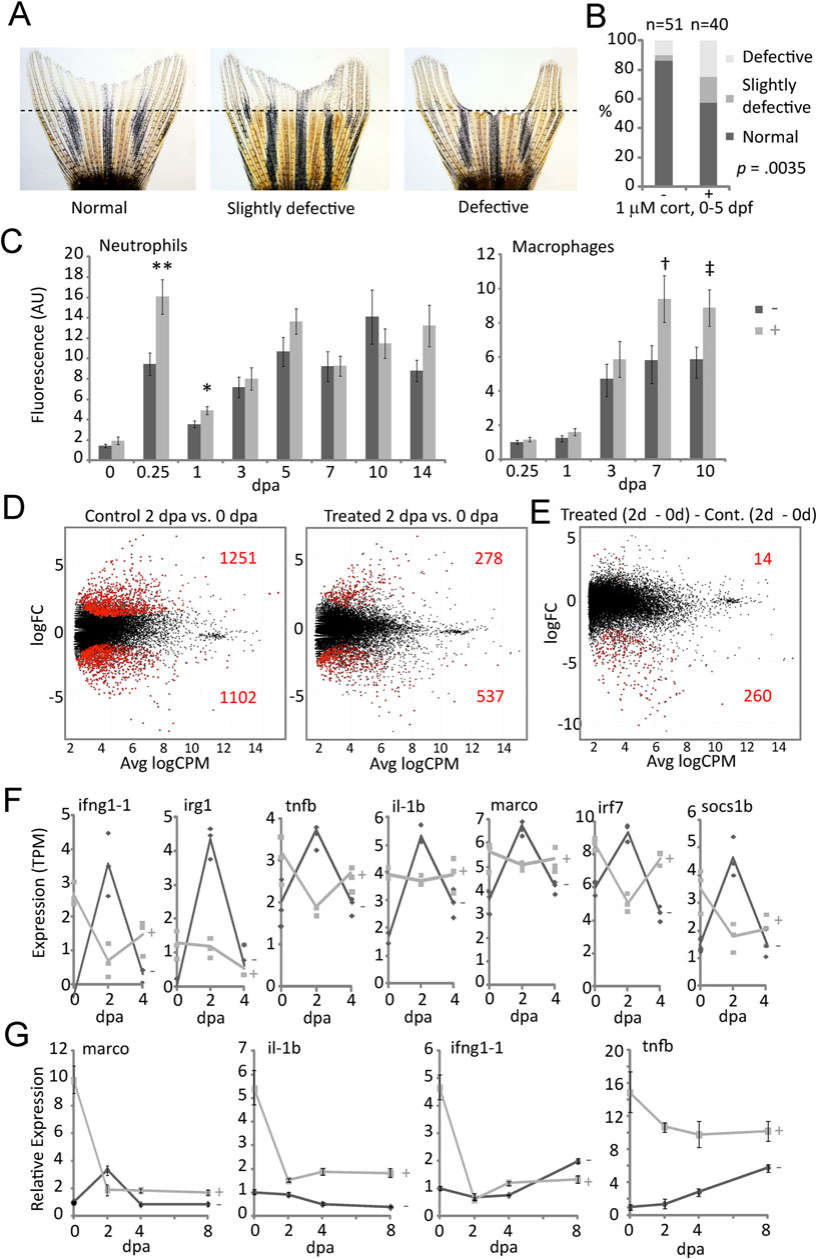
**Effects of early developmental exposure to cortisol on adult tailfin regeneration.** (A) Examples of morphologically normal, slightly defective and defective tailfin regeneration. (B) Quantification of combined data on regeneration morphology from three different experimental replicates from a single parental stock. Statistical significance was determined by a Chi-squared test (d.f.=1, comparing the total normal versus the total defective and slightly defective). Subsequent experiments using a newly imported parental stock did not show any overt morphological defects (possibly owing to the high polymorphism of the wild-type AB strain); however they did manifest the effects shown in panels C-F. (C) Quantification of neutrophils and macrophages in regenerating fin tissue (dpa, days post-amputation), respectively measured by fluorescence intensity of fins from *mpx:GFP* and *mpeg1:YFP* transgenic fish derived from untreated control embryos (−) or embryos treated with 1 μM cortisol (+). In one experiment counts of individual cells at a single time point confirmed that the differences in fluorescence intensity reflect differences in cell number. The bars represent the grand means±s.e.m. of measurements from three experimental replicates of the neutrophil counts, and two experimental replicates of the macrophage counts (a third macrophage experiment which did not show significant differences was not included, as it did not include the same time points). For the neutrophils the number of fish in each replicate ranged from 9-18, with overall totals of 41 control and 37 treated. For the macrophages the number of fish in each replicate ranged from 9-11, with overall totals of 21 control and 18 treated. Statistical significance **P=0.0017; *P=0.01; †*P*=0.028; ‡*P*=0.0226, by a two tailed *t*-test. (D) Scatter plots of average expression level and fold-change in expression for each gene in tailfin regenerates at 2 dpa compared to 0 dpa in 4.5-month-old control (untreated) fish and fish derived from cortisol-treated embryos. The red dots indicate genes with significant differential expression (*P*<0.05), the numbers of which (over- and under-expressed) are also shown in red. (E) Scatter plot of average expression level and fold-change in expression, showing interaction of cortisol treatment and time (0-2 dpa). Red dots indicate genes with significant differential expression (*P*<0.05), the numbers of which (over- and under-expressed) are also shown in red. (F) RNA-seq time course measurements of seven of the most significantly differentially expressed genes from the analysis shown in E; lines show the averages of three biological replicates, each of which are indicated by individual data points for both cortisol treatment (+, light gray) and untreated control (−, dark gray) groups. (G) qRT-PCR time course measurements of four of the genes shown in (F), during tailfin regeneration in 10 month old adults from the same cohort shown in D-F.

In a tailfin regeneration assay, several cohorts of adults in the cortisol-treated groups were found to produce a significantly higher frequency of morphological defects, ranging from mild patterning defects to complete failure of parts of the fin to regenerate (Fig. 3A,B; note caveat in the figure legend). Given the results described above and the recent report that macrophages play an essential role in adult tailfin regeneration (Petrie et al., 2014), we reasoned that the observed regeneration defects might reflect an aberrant inflammatory response to injury. To begin to assess whether this might be so we employed *mpx:GFP* (Mathias et al., 2006) and *mpeg1:YFP* (Roca and Ramakrishnan, 2013) transgenic lines to examine the respective dynamics of neutrophil and macrophage infiltration during tailfin regeneration. In fish from the cortisol-treated groups, greater numbers of neutrophils were found to initially enter the wound site, which was ultimately followed by greater numbers of macrophages in the regenerating fin (Fig. 3C), suggesting that these fish mount an exaggerated and possibly prolonged inflammatory response to injury.

RNA-seq analysis of regenerating tailfins from 4.5-month-old fish revealed that at 2 days post-amputation (dpa; corresponding to blastema formation), significantly fewer genes were mobilized in regenerating tissue of fish derived from cortisol-treated embryos (815 vs 2353 in controls; Fig. 3D). Gene ontology BP term enrichment (GOrilla) analysis of the genes differentially expressed between 2 and 0 dpa (Tables S5 and S6) gave strikingly contrasting results for fins from treated and control fish; the upregulated set from the controls was enriched with genes annotated with the BP parent categories *cell division*, *nuclear division*, *regeneration* (including *fin regeneration*), *response to stress*, *leukocyte migration*, and *immune system process*, whereas genes annotated in those categories were not enriched in the upregulated set of the cortisol-treated group (Fig. S7). Rather, genes annotated in categories associated with the immune system were instead enriched in the downregulated set of the cortisol-treated group (Fig. S8). This result was confirmed by comparing the interaction between treatment and regeneration time-course, which revealed that compared to controls, 274 genes were significantly differentially expressed in the fin tissue from treated fish at 2 dpa vs 0 dpa, with 260 of these being significantly under-expressed (Fig. 3E), many of them associated with the immune system defense response and/or its regulation (Fig. S9, Table S7). Plotting the temporal profiles of several of these genes revealed that they were highly but transiently upregulated at 2 dpa in regenerating fins of control fish, whereas their expression in treated fish was inverted; basally elevated compared to controls in the pre-regenerative tailfin (day 0), then transiently decreased at 2 dpa in the regenerating fin before becoming elevated again with respect to controls at 4 dpa (Fig. 3F). A similar pattern was observed on a global level when RNA-seq data from control and treated groups were compared at each time point and the results subjected to GOrilla analysis (Tables S8-S10): genes associated with *immune system process* and other categories related to immune system functions were basally over-expressed in the taifin, but under-expressed in the regenerating tailfin at 2 dpa, then again over-expressed at 4 dpa (Fig. S10). Quantitative RT-PCR measurements of *marco*, *il-1b*, *infg1-1* and *tnfb* from the same cohort of fish six months later confirmed that tailfin expression of these pro-inflammatory genes in the treated fish is basally elevated, then sharply downregulated during regeneration, inverse of their expression in untreated controls (Fig. 3G).

**Fig. 4.**
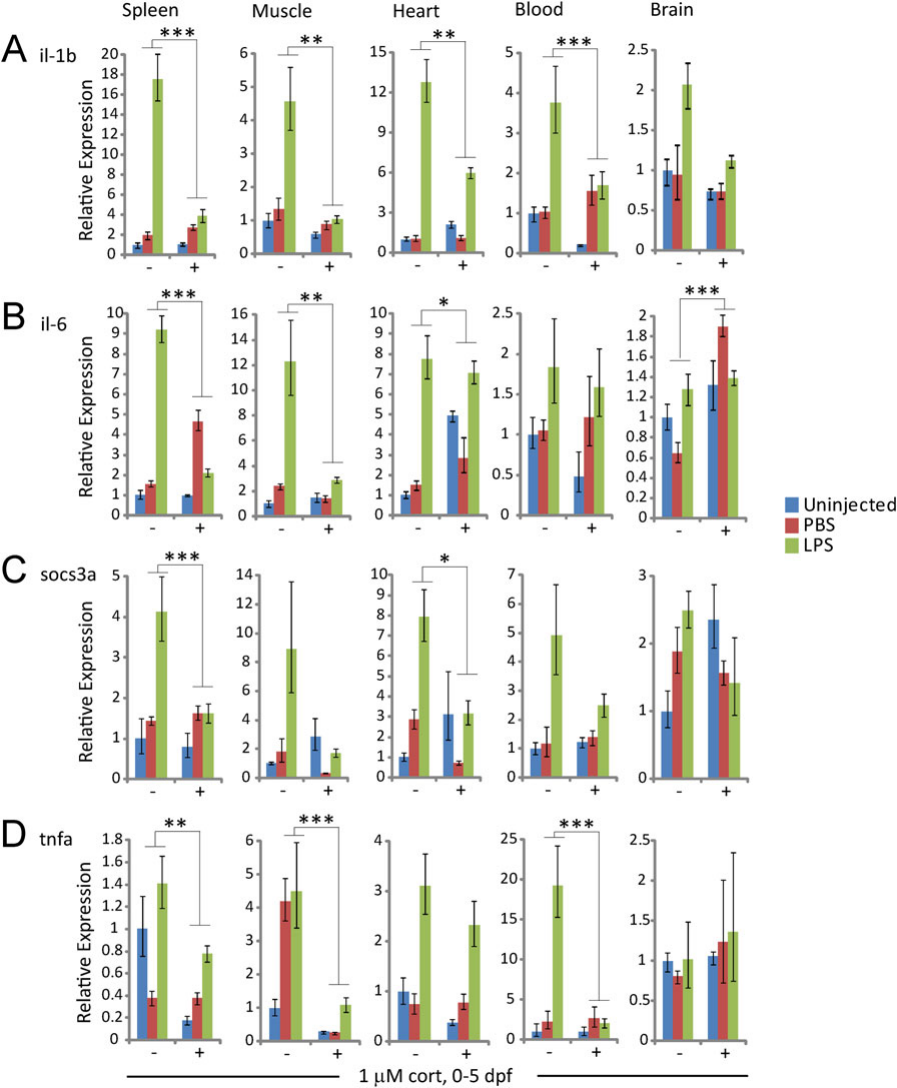
**Effects of early developmental exposure to cortisol on adult immune response to LPS.** Expression of (A) *il-1b*, (B) *il-6*, (C) *socs3a* and (D) *tnfa* in different tissues of adult fish derived from untreated (−) or cortisol-treated (+) embryos, in both uninjected controls and in fish injected 24 h previously with either PBS or LPS. Statistical significance was determined by four-way ANOVA with two treatments, LPS (compared to PBS) and cortisol; significant interaction between LPS and cortisol treatments are indicated as *P<0.05, **P<0.01, ***P<0.001, the error bars represent s.d. The experiment was repeated in a different cohort of experimental fish with similar results.

The results described above suggested that adult fish derived from cortisol-treated embryos have both aberrant basal expression of immune system genes and an impeded immune response. To further test this we injected adult fish with lipopolysaccharide (LPS), and used qRT-PCR to assay the expression of the LPS-responsive pro-inflammatory LPS-TLR4-responsive genes *il-1b*, *il6* and *tnfa* as well as the il-6-responsive immunoregulatory gene *socs3a* in peripheral tissues (spleen, skeletal muscle and heart), blood and brain. As expected, in control fish all four genes were strongly and specifically induced by LPS, albeit to different degrees in different tissues (Fig. 4). In contrast, in fish derived from cortisol-treated embryos LPS-induced expression of these genes was much weaker, in many cases being no different (or lower) than the expression induced non-specifically by PBS; in addition, basal expression of *il-1b*, *il-6* and *socs3a* was elevated in the heart (Fig. 4). Thus, chronic elevation of glucocorticoid signaling during early development has long term effects that increase the basal expression and/or non-specific induction of pro-inflammatory genes in adult tissues, while suppressing their specific induction in response to pathogenic challenge.

Collectively these data show that cortisol-treated embryos develop into adults wherein normal regulation of the immune system and inflammatory response is compromised. Our results further suggest that chronically elevated glucocorticoid signaling during early development reduces regenerative capacity of the adult. Although a previous report showed that synthetic glucocorticoids (but not cortisol) impair tailfin regeneration in larvae independently of their anti-inflammatory effects (Mathew et al., 2007), that study did not address the long-term effects of chronically elevated glucocorticoid signaling during early development on tailfin regeneration in adults, which is a very different context not directly comparable to that of the larva. It is thus possible that the regeneration defects that we observed were a consequence of the aberrant immunoregulation in the cortisol-treatment group, consistent with the emerging consensus that regulation of immune system activity is a critical determinant of adult somatic regenerative capacity (Forbes and Rosenthal, 2014). The observed effects on immunoregulation could stem from effects that elevated glucocorticoid signaling had on the developmental specification of immune cell fate, and/or from glucocorticoid-induced epigenetic changes that reduce the sensitivity of immune genes to glucocorticoids or other immunoregulatory signals. While further studies are needed to explore these possibilities, the findings reported here indicate that the zebrafish will provide a useful experimental model for elucidating the glucocorticoid-responsive gene regulatory networks and epigenetic mechanisms through which chronic early life stress affects adult immunoregulation.

## MATERIALS AND METHODS

### Zebrafish strains, husbandry and treatments

Unless otherwise noted, all experiments were performed using the AB strain of zebrafish. The *SR4G* transgenic reporter line (Krug et al., 2014) was kindly provided by Dr Karl Clark of the Mayo Clinic. The *mpeg1:YFP* (Roca and Ramakrishnan, 2013) and *mpx:GFP* (Mathias et al., 2006) reporter lines were provided by Dr Voot Yin of the MDI Biological Laboratory. The NF-κB-responsive GFP reporter line (Kanther et al., 2011) was provided by Dr Sandra Rieger of the MDI Biological Laboratory. Zebrafish were maintained in the animal facility of the MDI Biological Laboratory, in a recirculating system with a water temperature of 28.5°C, conductivity of 600-700 microsemens, pH of 7.2. Lights are timed on a 14 h light/10 h dark cycle. Matings and embryo/larva culture were carried out using standard procedures (Nasiadka and Clark, 2012). Embryos and larvae were cultured in plastic Petri dishes from 0-5 days post-fertilization (dpf). For cortisol treatments, stock solutions of cortisol-21-hemisuccinate sodium salt (Sigma H4881) in DMSO were added to the larval medium to achieve the indicated final concentrations; controls consisted of larvae in medium with an equivalent amount of DMSO vehicle. Cortisol and vehicle were replaced daily with media changes. At 5 dpf the fish were transferred to system tanks without water flow. Water flow began at 10 dpf. Fish were kept at a density of 30 fish/liter until 1 month post-fertilization, then were split up to a density of 5 fish/liter maximum. From 5-14 dpf the fish were fed O.S.I. Rotofier Diet three times per day. From 15-21 dpf the fish were fed Golden Pearl Active Spheres (Brine Shrimp Direct) 50-100 μm three times per day. From 22-60 dpf the fish were fed a 50:50 mix of Golden Pearl Active Spheres 100-200 μm and Golden Pearl Active Spheres 200-300 μm twice a day, and once a day with Brine Shrimp Direct Spirulina & Kelp Flake. Adults are fed once per day with live artemia, hatched daily and 1× per day with a 50:50 mixture of Golden Pearl Active Spheres 800-1000 μM and Aquaneering Fish Food Zebrafish Select Diet.

### Caudal fin amputations and regeneration

Adult zebrafish were anesthetized in tricaine and caudal fins were amputated halfway between the edge of the caudal peduncle and the distal tip of the fin, using a razor blade. Regeneration progress was monitored at post-amputation time points using an Olympus MVX10 fluorescence stereomicroscope. Fin areas were quantified using ImageJ (NIH).

### LPS injection

Adult zebrafish were fasted for 24 h, and then anesthetized in tricaine. Fish were injected intraperitoneally with 10 μl 1 mg/ml lipopolysaccharide (Sigma L3012) in PBS or PBS only, using a 10 μl 701-LT Hamilton syringe fitted with a 35-gauge needle. Fish were then transferred immediately back into system water and fasted for an additional 24 h before tissue collection.

### Tissue collection

Adult zebrafish were euthanized in tricaine and blood was collected using the method described by Babaei et al. (2013). Other tissues were dissected out and snap frozen in liquid nitrogen. All procedures were approved by the Institutional Animal Care and Use Committee (IACUC).

### Cortisol measurements

Larvae were washed of treatment media and pooled (*n*=30), then snap frozen in liquid nitrogen. Adult zebrafish were euthanized in tricaine and snap frozen in liquid nitrogen and frozen tissues were minced with a razor. Samples were homogenized in PBS using an automatic pestle grinder. A liquid-liquid extraction was performed with ethyl acetate and the organic, cortisol-containing phase was collected. The solvent was evaporated under a stream of nitrogen gas, and the cortisol extract was re-dissolved in Cortisol ELISA Extraction Buffer (Neogen Corporation). Samples were assayed for cortisol content using the Neogen Cortisol ELISA kit and quantified with a standard curve.

### ROS measurements

Zebrafish larvae were incubated in 10 μM CM-H_2_DCFDA (Molecular Probes/Life Technologies C6827) in embryo water at 28°C for 1 h, 15 min under darkness. Larvae were anesthetized in tricaine and imaged using an Olympus MVX10 fluorescence stereomicroscope. Fluorescence intensity was quantified using ImageJ software (NIH).

### Fluorescence imaging

Fluorescently labeled zebrafish larvae and adults were imaged using an Olympus MVX10 fluorescence stereomicroscope, following their anesthetization in tricaine. Fluorescence data were quantified using NIH ImageJ software, as integrated pixel intensity within a defined region of interest (ROI), minus the average background pixel intensity times the ROI area.

### RNA-seq

Total RNA was extracted from 5-day larvae (30 per each of three biological replicates) or dissected tissues from adults (5 adults per each of three biological replicates) using the RNA-EZplus kit from Qiagen, and quantified using a Nanodrop spectrophotometer. Indexed strand-specific polyA+-selected mRNA libraries were prepared and paired-end sequenced on an Illumina HiSeq2500 at the HudsonAlpha Institute for Biotechnology (Birmingham, AL) following manufacturer's protocols. Following sequence read quality control diagnostic analyses using FastQC version 0.11.2 (http://www.bioinformatics.babraham.ac.uk/projects/fastqc/), reads were trimmed using Trimmomatic version 0.32 (Bolger et al., 2014). Trimmed reads from the larval libraries were aligned to the zebrafish (*Danio rerio*) genome assembly version Zv9 using TopHat version 2.0.11 (Trapnell et al., 2009) and read counts per gene generated using HTSeq version 0.6.1 (Anders et al., 2015). The larval RNA-seq data were deposited in the National Center for Biotechnology Information (NCBI) Gene Expression Omnibus (GEO) database, under accession number GSE80221. The trimmed reads from the 4.5-month-old adult libraries were aligned to the annotated transcriptome from Ensembl version 83 (Flicek et al., 2014) using RSEM version 1.2.25 (Li and Dewey, 2011) and Bowtie 1.1.2 (Langmead et al., 2009). Read counts per gene from either HTSeq or RSEM were analyzed using R statistical computing environment (http://r-project.org) version 3.2.1 and R/edgeR version 3.2.1 (Robinson et al., 2010). The adult RNA-seq data were deposited in the NCBI GEO database, under accession number GSE80260. Both larval and adult data sets can be accessed via the GEO SuperSeries, under accession number GSE80286.

### Gene ontology enrichment analysis

Gene ontology enrichment analysis was performed using PANTHER (Mi et al., 2013) or GOrilla (Eden et al., 2009), using either a single ranked list or two unranked lists (target and background) of genes, as described in the Results and figure legends for each analysis. Enriched terms found by GOrilla were visualized using REVIGO (Supek et al., 2011).

### Measurement of gene expression by quantitative PCR

RNA prepared from whole larvae (30 larvae) or dissected adult tissues (from five fish, determined by a power analysis to be sufficient for statistical significance given measured effect sizes) as described above was converted to cDNA using Superscript III (Life Technologies), and transcript levels were quantified by quantitative real-time PCR (qPCR) measurement of SYBR Green fluorescence using Quanta Biosciences PerfeCTa FastMix on a Roche Lightcycler 480II. Primer sequences are shown in Table 2. Relative levels of expression were calculated using the delta-delta Ct method, using the average of Cts obtained for beta-actin and RPL13a for normalization.

**Table 2.**
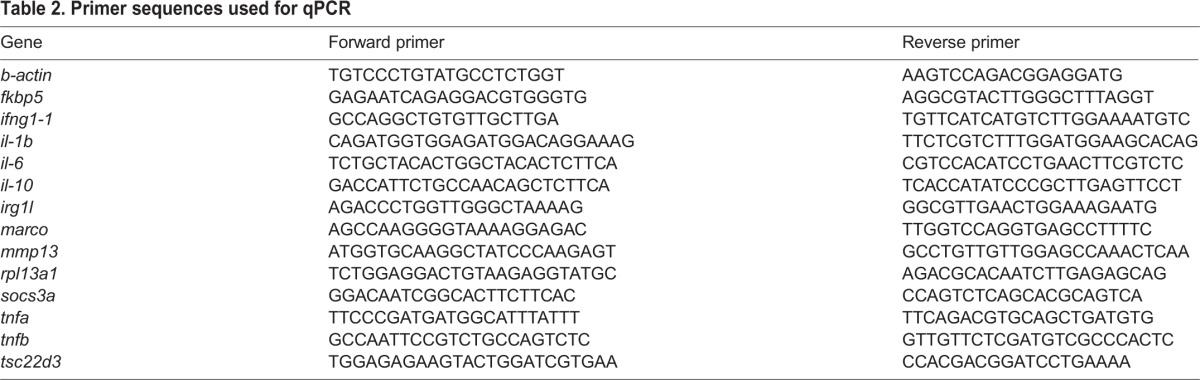
**Primer sequences used for qPCR**

## References

[BIO020065C1] Anders S., Pyl P. T. and Huber W. (2015). HTSeq–a Python framework to work with high-throughput sequencing data. *Bioinformatics* 31, 166-169. 10.1093/bioinformatics/btu638 25260700 PMC4287950

[BIO020065C49] Babaei F., Ramalingam R., Tavendale A., Liang Y., Yan L. S., Ajuh P., Cheng S. H. and Lam Y. W. (2013). Novel blood collection method allows plasma proteome analysis from single zebrafish. *J. Proteome Res.* 12, 1580-1590. 10.1021/pr3009226 23413775

[BIO020065C2] Berk M., Williams L. J., Jacka F. N., O'Neil A., Pasco J. A., Moylan S., Allen N. B., Stuart A. L., Hayley A. C., Byrne M. L. et al. (2013). So depression is an inflammatory disease, but where does the inflammation come from? *BMC Med.* 11, 200 10.1186/1741-7015-11-200 24228900 PMC3846682

[BIO020065C3] Bolger A. M., Lohse M. and Usadel B. (2014). Trimmomatic: a flexible trimmer for Illumina sequence data. *Bioinformatics* 30, 2114-2120. 10.1093/bioinformatics/btu170 24695404 PMC4103590

[BIO020065C4] Buckley C. D., Gilroy D. W. and Serhan C. N. (2014). Proresolving lipid mediators and mechanisms in the resolution of acute inflammation. *Immunity* 40, 315-327. 10.1016/j.immuni.2014.02.009 24656045 PMC4004957

[BIO020065C5] Chen P., Jiang T., Ouyang J., Cui Y. and Chen Y. (2009). Epigenetic programming of diverse glucocorticoid response and inflammatory/immune-mediated disease. *Med. Hypotheses* 73, 657-658. 10.1016/j.mehy.2009.08.013 19726136

[BIO020065C6] Chinenov Y., Coppo M., Gupte R., Sacta M. A. and Rogatsky I. (2014). Glucocorticoid receptor coordinates transcription factor-dominated regulatory network in macrophages. *BMC Genomics* 15, 656 10.1186/1471-2164-15-656 25099603 PMC4133603

[BIO020065C7] Christian L. M., Graham J. E., Padgett D. A., Glaser R. and Kiecolt-Glaser J. K. (2006). Stress and wound healing. *Neuroimmunomodulation* 13, 337-346. 10.1159/000104862 17709956 PMC2792763

[BIO020065C8] Cohen S., Janicki-Deverts D. and Miller G. E. (2007). Psychological stress and disease. *JAMA* 298, 1685-1687. 10.1001/jama.298.14.1685 17925521

[BIO020065C9] Cohen S., Janicki-Deverts D., Doyle W. J., Miller G. E., Frank E., Rabin B. S. and Turner R. B. (2012). Chronic stress, glucocorticoid receptor resistance, inflammation, and disease risk. *Proc. Natl. Acad. Sci. USA* 109, 5995-5999. 10.1073/pnas.1118355109 22474371 PMC3341031

[BIO020065C10] Drake A. J., Tang J. I. and Nyirenda M. J. (2007). Mechanisms underlying the role of glucocorticoids in the early life programming of adult disease. *Clin. Sci.* 113, 219-232. 10.1042/CS20070107 17663659

[BIO020065C11] Eden E., Navon R., Steinfeld I., Lipson D. and Yakhini Z. (2009). GOrilla: a tool for discovery and visualization of enriched GO terms in ranked gene lists. *BMC Bioinformatics* 10, 48 10.1186/1471-2105-10-48 19192299 PMC2644678

[BIO020065C12] Ellett F., Pase L., Hayman J. W., Andrianopoulos A. and Lieschke G. J. (2011). mpeg1 promoter transgenes direct macrophage-lineage expression in zebrafish. *Blood* 117, e49-e56. 10.1182/blood-2010-10-314120 21084707 PMC3056479

[BIO020065C13] Flicek P., Amode M. R., Barrell D., Beal K., Billis K., Brent S., Carvalho-Silva D., Clapham P., Coates G., Fitzgerald S. et al. (2014). Ensembl 2014. *Nucleic Acids Res.* 42, D749-D755. 10.1093/nar/gkt1196 24316576 PMC3964975

[BIO020065C14] Forbes S. J. and Rosenthal N. (2014). Preparing the ground for tissue regeneration: from mechanism to therapy. *Nat. Med.* 20, 857-869. 10.1038/nm.3653 25100531

[BIO020065C15] Godwin J. W., Pinto A. R. and Rosenthal N. A. (2013). Macrophages are required for adult salamander limb regeneration. *Proc. Natl. Acad. Sci. USA* 110, 9415-9420. 10.1073/pnas.1300290110 23690624 PMC3677454

[BIO020065C16] Gomez-Sanchez E. and Gomez-Sanchez C. E. (2014). The multifaceted mineralocorticoid receptor. *Compr. Physiol.* 4, 965-994. 10.1002/cphy.c130044 24944027 PMC4521600

[BIO020065C17] Hall C. J., Boyle R. H., Sun X., Wicker S. M., Misa J. P., Krissansen G. W., Print C. G., Crosier K. E. and Crosier P. S. (2014). Epidermal cells help coordinate leukocyte migration during inflammation through fatty acid-fuelled matrix metalloproteinase production. *Nat. Commun.* 5, 3880 10.1038/ncomms4880 24852213

[BIO020065C18] Harris A. and Seckl J. (2011). Glucocorticoids, prenatal stress and the programming of disease. *Horm. Behav.* 59, 279-289. 10.1016/j.yhbeh.2010.06.007 20591431

[BIO020065C19] Howerton C. L. and Bale T. L. (2012). Prenatal programing: at the intersection of maternal stress and immune activation. *Horm. Behav.* 62, 237-242. 10.1016/j.yhbeh.2012.03.007 22465455 PMC3568743

[BIO020065C20] Jung S. H., Wang Y., Kim T., Tarr A., Reader B., Powell N. and Sheridan J. F. (2015). Molecular mechanisms of repeated social defeat-induced glucocorticoid resistance: Role of microRNA. *Brain Behav. Immun.* 44, 195-206. 10.1016/j.bbi.2014.09.015 25317829 PMC4275324

[BIO020065C21] Kadmiel M. and Cidlowski J. A. (2013). Glucocorticoid receptor signaling in health and disease. *Trends Pharmacol. Sci.* 34, 518-530. 10.1016/j.tips.2013.07.003 23953592 PMC3951203

[BIO020065C22] Kanther M., Sun X., Mühlbauer M., Mackey L. C., Flynn E. J. III, Bagnat M., Jobin C. and Rawls J. F. (2011). Microbial colonization induces dynamic temporal and spatial patterns of NF-kappaB activation in the zebrafish digestive tract. *Gastroenterology* 141, 197-207. 10.1053/j.gastro.2011.03.042 21439961 PMC3164861

[BIO020065C23] Khulan B. and Drake A. J. (2012). Glucocorticoids as mediators of developmental programming effects. *Best Pract. Res. Clin. Endocrinol. Metab.* 26, 689-700. 10.1016/j.beem.2012.03.007 22980050

[BIO020065C24] Krug R. G. II, Poshusta T. L., Skuster K. J., Berg M. R., Gardner S. L. and Clark K. J. A. (2014). A transgenic zebrafish model for monitoring glucocorticoid receptor activity. *Genes Brain Behav.* 13, 478-487. 10.1111/gbb.12135 24679220 PMC4177977

[BIO020065C25] Langmead B., Trapnell C., Pop M. and Salzberg S. L. (2009). Ultrafast and memory-efficient alignment of short DNA sequences to the human genome. *Genome Biol.* 10, R25 10.1186/gb-2009-10-3-r25 19261174 PMC2690996

[BIO020065C26] Li B. and Dewey C. N. (2011). RSEM: accurate transcript quantification from RNA-Seq data with or without a reference genome. *BMC Bioinformatics* 12, 323 10.1186/1471-2105-12-323 21816040 PMC3163565

[BIO020065C27] Maldonado Bouchard S. and Hook M. A. (2014). Psychological stress as a modulator of functional recovery following spinal cord injury. *Front. Neurol.* 5, 44 10.3389/fneur.2014.00044 24782818 PMC3988397

[BIO020065C28] Mathew L. K., Sengupta S., Kawakami A., Andreasen E. A., Löhr C. V., Loynes C. A., Renshaw S. A., Peterson R. T. and Tanguay R. L. (2007). Unraveling tissue regeneration pathways using chemical genetics. *J. Biol. Chem.* 282, 35202-35210. 10.1074/jbc.M706640200 17848559

[BIO020065C29] Mathias J. R., Perrin B. J., Liu T. -X., Kanki J., Look A. T. and Huttenlocher A. (2006). Resolution of inflammation by retrograde chemotaxis of neutrophils in transgenic zebrafish. *J. Leukoc. Biol.* 80, 1281-1288. 10.1189/jlb.0506346 16963624

[BIO020065C30] Mi H., Muruganujan A., Casagrande J. T. and Thomas P. D. (2013). Large-scale gene function analysis with the PANTHER classification system. *Nat. Protoc.* 8, 1551-1566. 10.1038/nprot.2013.092 23868073 PMC6519453

[BIO020065C31] Miller G. E., Chen E., Fok A. K., Walker H., Lim A., Nicholls E. F., Cole S. and Kobor M. S. (2009). Low early-life social class leaves a biological residue manifested by decreased glucocorticoid and increased proinflammatory signaling. *Proc. Natl. Acad. Sci. USA* 106, 14716-14721. 10.1073/pnas.0902971106 19617551 PMC2732821

[BIO020065C32] Nasiadka A. and Clark M. D. (2012). Zebrafish breeding in the laboratory environment. *ILAR J.* 53, 161-168. 10.1093/ilar.53.2.161 23382347

[BIO020065C33] Nayak L., Goduni L., Takami Y., Sharma N., Kapil P., Jain M. K. and Mahabeleshwar G. H. (2013). Kruppel-like factor 2 is a transcriptional regulator of chronic and acute inflammation. *Am. J. Pathol.* 182, 1696-1704. 10.1016/j.ajpath.2013.01.029 23499374 PMC3644709

[BIO020065C34] Nesan D. and Vijayan M. M. (2013). Role of glucocorticoid in developmental programming: evidence from zebrafish. *Gen. Comp. Endocrinol.* 181, 35-44. 10.1016/j.ygcen.2012.10.006 23103788

[BIO020065C35] Pawelec G., Goldeck D. and Derhovanessian E. (2014). Inflammation, ageing and chronic disease. *Curr. Opin. Immunol.* 29, 23-28. 10.1016/j.coi.2014.03.007 24762450

[BIO020065C36] Petrie T. A., Strand N. S., Tsung-Yang C., Rabinowitz J. S. and Moon R. T. (2014). Macrophages modulate adult zebrafish tail fin regeneration. *Development* 141, 2581-2591. 10.1242/dev.098459 24961798 PMC4067955

[BIO020065C37] Qin H., Holdbrooks A. T., Liu Y., Reynolds S. L., Yanagisawa L. L. and Benveniste E. N. (2012). SOCS3 deficiency promotes M1 macrophage polarization and inflammation. *J. Immunol.* 189, 3439-3448. 10.4049/jimmunol.1201168 22925925 PMC4184888

[BIO020065C38] Radek K. A. (2010). Antimicrobial anxiety: the impact of stress on antimicrobial immunity. *J. Leukoc. Biol.* 88, 263-277. 10.1189/jlb.1109740 20442225 PMC2908944

[BIO020065C39] Robinson M. D., McCarthy D. J. and Smyth G. K. (2010). edgeR: a Bioconductor package for differential expression analysis of digital gene expression data. *Bioinformatics* 26, 139-140. 10.1093/bioinformatics/btp616 19910308 PMC2796818

[BIO020065C40] Roca F. J. and Ramakrishnan L. (2013). TNF dually mediates resistance and susceptibility to mycobacteria via mitochondrial reactive oxygen species. *Cell* 153, 521-534. 10.1016/j.cell.2013.03.022 23582643 PMC3790588

[BIO020065C41] Sorrells S. F. and Sapolsky R. M. (2007). An inflammatory review of glucocorticoid actions in the CNS. *Brain Behav. Immun.* 21, 259-272. 10.1016/j.bbi.2006.11.006 17194565 PMC1997278

[BIO020065C42] Stark J. L., Avitsur R., Padgett D. A., Campbell K. A., Beck F. M. and Sheridan J. F. (2001). Social stress induces glucocorticoid resistance in macrophages. *Am. J. Physiol. Regul. Integr. Comp. Physiol.* 280, R1799-R1805. 11353685 10.1152/ajpregu.2001.280.6.R1799

[BIO020065C43] Su T. and Ding X. (2004). Regulation of the cytochrome P450 2A genes. *Toxicol. Appl. Pharmacol.* 199, 285-294. 10.1016/j.taap.2003.11.029 15364544

[BIO020065C44] Supek F., Bošnjak M., Škunca N. and Šmuc T. (2011). REVIGO summarizes and visualizes long lists of gene ontology terms. *PLoS ONE* 6, e21800 10.1371/journal.pone.0021800 21789182 PMC3138752

[BIO020065C45] Trapnell C., Pachter L. and Salzberg S. L. (2009). TopHat: discovering splice junctions with RNA-Seq. *Bioinformatics* 25, 1105-1111. 10.1093/bioinformatics/btp120 19289445 PMC2672628

[BIO020065C46] Tyrka A. R., Price L. H., Marsit C., Walters O. C. and Carpenter L. L. (2012). Childhood adversity and epigenetic modulation of the leukocyte glucocorticoid receptor: preliminary findings in healthy adults. *PLoS ONE* 7, e30148 10.1371/journal.pone.0030148 22295073 PMC3266256

[BIO020065C47] Vago J. P., Nogueira C. R. C., Tavares L. P., Soriani F. M., Lopes F., Russo R. C., Pinho V., Teixeira M. M. and Sousa L. P. (2012). Annexin A1 modulates natural and glucocorticoid-induced resolution of inflammation by enhancing neutrophil apoptosis. *J. Leukoc. Biol.* 92, 249-258. 10.1189/jlb.0112008 22493082

[BIO020065C48] Weaver I. C. G., Cervoni N., Champagne F. A., D'Alessio A. C., Sharma S., Seckl J. R., Dymov S., Szyf M. and Meaney M. J. (2004). Epigenetic programming by maternal behavior. *Nat. Neurosci.* 7, 847-854. 10.1038/nn1276 15220929

